# Preparations and Properties of Ionic Liquid-Assisted Electrospun Biodegradable Polymer Fibers

**DOI:** 10.3390/polym14122308

**Published:** 2022-06-07

**Authors:** Ahmad Adlie Shamsuri, Khalina Abdan, Siti Nurul Ain Md. Jamil

**Affiliations:** 1 Laboratory of Biocomposite Technology, Institute of Tropical Forestry and Forest Products, Universiti Putra Malaysia (UPM), Serdang 43400, Selangor, Malaysia; 2 Department of Chemistry, Faculty of Science, Universiti Putra Malaysia (UPM), Serdang 43400, Selangor, Malaysia; ctnurulain@upm.edu.my; 3 Centre of Foundation Studies for Agricultural Science, Universiti Putra Malaysia (UPM), Serdang 43400, Selangor, Malaysia

**Keywords:** ionic liquid, electrospinning, biodegradable, polymer, fiber

## Abstract

Enhanced awareness of the environment and environmental conservation has inspired researchers to search for replacements for the use of volatile organic compounds in the processing of polymers. Recently, ionic liquids have been utilized as solvents for solvating natural and synthetic biodegradable polymers since they are non-volatile, recyclable, and non-flammable. They have also been utilized to prepare electrospun fibers from biodegradable polymers. In this concise review, examples of natural and synthetic biodegradable polymers that are generally employed as materials for the preparation of electrospun fibers are shown. In addition, examples of ionic liquids that are utilized in the electrospinning of biodegradable polymers are also displayed. Furthermore, the preparations of biodegradable polymer electrospinning solutions utilizing ionic liquids are demonstrated. Additionally, the properties of electrospun biodegradable polymer fibers assisted by different ionic liquids are also concisely reviewed. Besides this, the information acquired from this review provides a much deeper understanding of the preparation of electrospinning solutions and the essential properties of electrospun biodegradable polymer fibers. In summary, this concise review discovered that different functions (solvent or additive) of ionic liquids could provide distinct properties to electrospun fibers.

## 1. Introduction

Electrospun biodegradable polymer fibers are made from biodegradable polymers via the electrospinning method. Electrospinning consumes a high-voltage electric field to eject micron and nano-sized fibers from a polymer solution through a charged spinneret towards the collecting electrode [1]. Electrospun biodegradable polymer fibers have become an attractive material to investigate because of their biocompatibility, biodegradability, and regenerative properties [2]. Furthermore, the thickness of electrospun fibers can be altered by adjusting the spinning parameters [3], such as the rheological properties of the polymer solution, needle/collector distance, type of collector, and voltage applied [4]. On top of that, there are two types of biodegradable polymers, specifically natural and synthetic polymers. Natural polymers are polymers that are obtained from plants or animals, whilst synthetic polymers are totally artificial. They can be divided into microbiologically made polymers, petroleum-based polymers, and polymers that are made from natural waste. Table 1 shows examples of natural and synthetic biodegradable polymers that were employed for the preparation of electrospun fibers. It can be observed that cellulose is frequently employed for this purpose. This is because cellulose is one of the most abundant biodegradable polymers on the earth.

**Figure 1:**
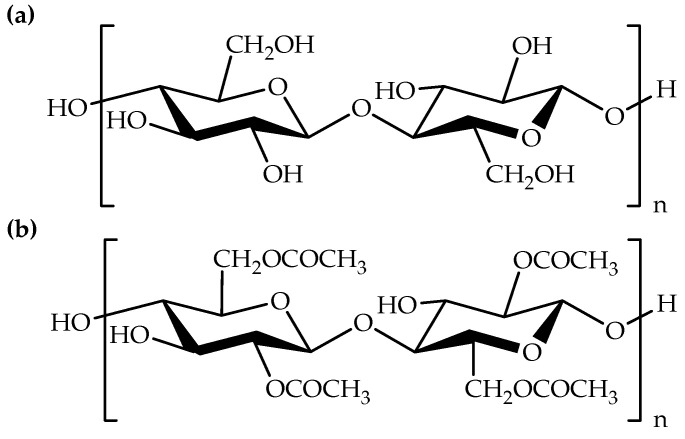

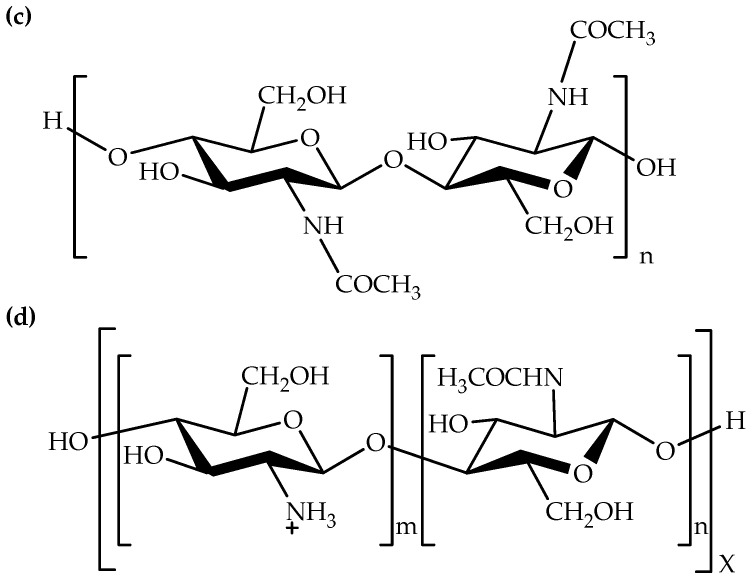
Chemical structures of (**a**) cellulose, (**b**) cellulose acetate, (**c**) chitin, and (**d**) chitosan.

**Figure 2:**
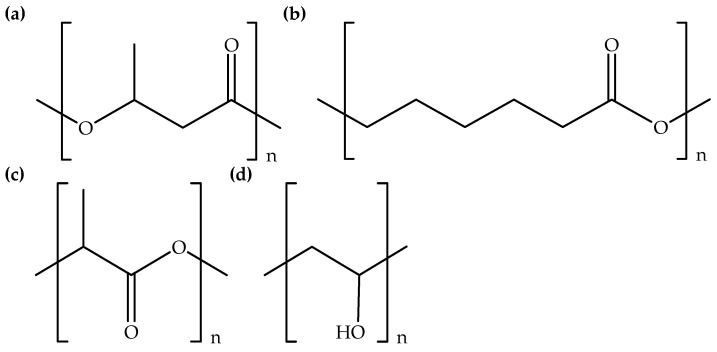
Chemical structures of (**a**) PHB, (**b**) PCL, (**c**) PLA, and (**d**) PVA.

Recently, chitin, the second most abundant natural polymer after cellulose [1,29] has also been employed for the preparation of electrospun fibers. In addition, cellulose and chitin derivatives, such as cellulose acetate [24,25,26] and chitosan [12,30] were employed to prepare electrospun fibers. Both biodegradable polymer derivatives have good solubility with conventional organic solvents, compared to their neat polymers. Figure 1 exhibits the chemical structures of cellulose, cellulose acetate, chitin, and chitosan. Besides this, it can also be seen in Table 1 that synthetic biodegradable polymers, for example, polyhydroxybutyrate (PHB); polycaprolactone (PCL); polylactic acid (PLA); and polyvinyl alcohol (PVA) can be employed for the preparation of electrospun fibers [3,4,29,34]. Moreover, previous studies have shown that natural–synthetic polymer hybrid fibers have also been prepared via the electrospinning method [29,34]. Figure 2 displays the chemical structures of PHB, PCL, PLA, and PVA. In general, synthetic biodegradable polymer solutions can be easily prepared using organic solvents, such as chloroform, tetrahydrofuran, etc., prior to the electrospinning process. Additionally, fluorinated alcohol like 1,1,1,3,3,3-hexafluoropropanol can also be used as a solvent in the preparation of synthetic biodegradable polymer solutions. On top of that, carboxylic acids, such as formic acid and acetic acid have been used as low-toxicity solvents for the electrospinning of natural biodegradable polymers [31].

Nevertheless, natural biodegradable polymers, for instance cellulose, chitin, and silk are insoluble in conventional organic solvents. Therefore, alternative solvents, such as ionic liquids have been utilized to dissolve the natural biodegradable polymers before the initiation of the electrospinning process. The utilization of ionic liquids in the dissolution of natural polymers is an excellent option as they are non-volatile, recyclable, and non-flammable. It is known that ionic liquids are molten salts that solely comprise cations and anions. They have low melting points (<100 °C), high thermal and chemical stability, and good electrical conductivity. Furthermore, ionic liquids are also designable and their structures can be tweaked by changing cations or anions [2]. Currently, ionic liquids are widely utilized in the dissolution and modification of natural and synthetic biodegradable polymers [40,41]. Therefore, the utilization of ionic liquids in the electrospinning of biodegradable polymers is encouraging because ionic liquids have an excellent dissolution ability, negligible vapor pressure, good ionic conductivity, and ease of solvent recovery [2,6,37].

Table 2 indicates examples of ionic liquids that were utilized in the electrospinning of biodegradable polymers. It can be seen that imidazolium-based ionic liquids with different counter anions are often utilized, compared to the ammonium-based ionic liquid. This is because they are effortlessly obtainable and they also have intriguing properties for use in numerous applications. Moreover, in the past decade, imidazolium-based ionic liquids have acted as solvents for natural and synthetic biodegradable polymers. However, they can also act as additives for the electrospinning of biodegradable polymers. In addition, until now and to the best knowledge of the authors, no concise reviews have been conducted concerning the preparation and properties of ionic liquid-assisted electrospun biodegradable polymer fibers. Thus, this is the purpose of this organized review which, albeit limited and in no way comprehensive, is still related to other current studies.

## 2. Ionic Liquids as Solvents and Additives for Electrospinning of Biodegradable Polymers

### 2.1. Ionic Liquids as Solvents for Electrospinning of Biodegradable Polymers

**Figure 3:**
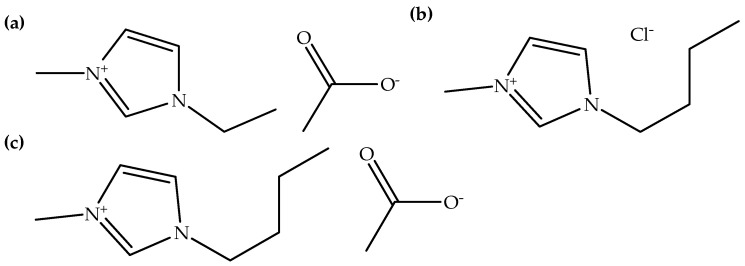
Chemical structures of (**a**) [C_2_mim][OAc], (**b**) [C_4_mim][Cl], and (**c**) [C_4_mim][OAc].

Ionic liquids have good solubility with many organic and inorganic solvents. They are also capable of dissolving most organic materials and some inorganic materials, including biodegradable polymers. Table 3 shows examples of ionic liquids that were utilized as solvents for the electrospinning of biodegradable polymers. It can be seen that imidazolium-based ionic liquids with acetate and chloride counter anions are typically utilized as solvents for the dissolution of biodegradable polymers. Additionally, [C_2_mim][OAc] is the main ionic liquid utilized as a solvent, followed by [C_4_mim][Cl] and [C_4_mim][OAc]. [C_2_mim][OAc] can be synthesized from [C_2_mim][Cl] via a metathesis reaction [41]. On the other hand, [C_4_mim][Cl] can be synthesized from 1-methylimidazole through an alkylation reaction [41]. Figure 3 exhibits the chemical structures of [C_2_mim][OAc], [C_4_mim][Cl], and [C_4_mim][OAc]. Moreover, it can also be seen in Table 3 that cellulose, silk, and chitin were mostly dissolved in ionic liquids, compared to other natural biodegradable polymers. In addition, synthetic biodegradable polymers, such as PCL and PLA can be dissolved in ionic liquids [4,29]. This proved that the ionic liquids could not only be utilized for dissolving natural polymers but also synthetic polymers.

### 2.2. Ionic Liquids as Additives for Electrospinning of Biodegradable Polymers

**Figure 4:**
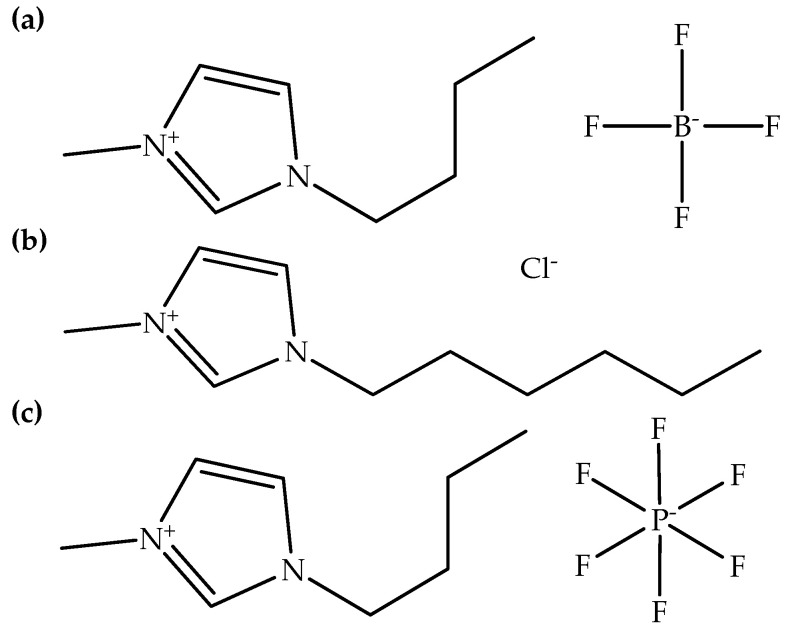
Chemical structures of (**a**) [C_4_mim][BF_4_], (**b**) [C_6_mim][Cl], and (**c**) [C_4_mim][PF_6_].

Ionic liquids can also be utilized as additives for electrospun biodegradable polymer fibers by adding a small amount into biodegradable polymer solutions before the electrospinning process. Table 4 demonstrates examples of ionic liquids that were utilized as additives for the electrospinning of biodegradable polymers. It can be found that imidazolium-based ionic liquids with tetrafluoroborate, chloride, and hexafluorophosphate counter anions are usually utilized as additives for electrospun fibers. In addition, [C_4_mim][BF_4_] is the most ionic liquid utilized as an additive, followed by [C_6_mim][Cl] and [C_4_mim][PF_6_]. [C_4_mim][BF_4_] and [C_4_mim][PF_6_] can be synthesized from [C_4_mim][Cl] via a metathesis reaction. In contrast, [C_6_mim][Cl] can be synthesized from 1-methylimidazole through an alkylation reaction same as [C_4_mim][Cl]. Figure 4 presents the chemical structures of [C_4_mim][BF_4_], [C_6_mim][Cl], and [C_4_mim][PF_6_]. Besides this, Table 4 also shows that synthetic biodegradable polymers were generally added with ionic liquids, compared to natural biodegradable polymers. Additionally, cellulose and chitin derivatives, for example, cellulose acetate, ethyl cellulose, and chitosan can be added with ionic liquids through their solutions in the preparation of electrospun fibers.

## 3. Preparations of Biodegradable Polymer Electrospinning Solutions

### 3.1. Ionic Liquids as Solvents for Preparing Electrospinning Solutions

Table 5 displays the ionic liquids, biodegradable polymers, biodegradable polymer concentrations, dissolution temperatures, and dissolution times that were applied for the preparation of electrospinning solutions. It can be observed that imidazolium-based ionic liquids with short alkyl chains, such as ethyl, allyl, and butyl are commonly utilized as solvents for preparing biodegradable polymer electrospinning solutions. Moreover, imidazolium-based ionic liquids with acetate and chloride counter anions are effectively utilized in the dissolution of biodegradable polymers. Besides this, natural biodegradable polymers, for example, cellulose, chitin, and silk are often employed for the preparation of electrospinning solutions. Furthermore, synthetic biodegradable polymer electrospinning solutions can also be prepared by utilizing ionic liquids [4,29]. In Table 5, it can also be seen that different biodegradable polymer concentrations were applied for preparing electrospinning solutions. The maximum concentration of cellulose in ionic liquid for an electrospinning solution was 10 wt.% [13,19], and the minimum was 1.2 wt.% [5]. On the other hand, the maximum concentration of chitin in ionic liquid for an electrospinning solution was 1.75 wt.% [29], and the minimum was 0.4 wt.% [9].

On the contrary, the maximum concentration of silk in ionic liquid for an electrospinning solution was 10 wt.% [36,37], and the minimum was 5 wt.% [19]. On top of that, the concentrations of PCL and PLA in ionic liquids for electrospinning solutions were 20 wt.% and 0.5 wt.%, respectively. Therefore, the distinct types of biodegradable polymers possess different concentrations in different ionic liquids. Nevertheless, this depends on the types of ionic liquids that are utilized for electrospinning solutions. Furthermore, other aspects that can influence the dissolution efficiency of biodegradable polymers in ionic liquids are dissolution temperature and dissolution time. It can also be observed in Table 5 that an increase in the dissolution temperature decreased the dissolution time of biodegradable polymers, especially for [C_4_mim][Cl] and [C_2_mim][OAc]. In this circumstance, the dissolution time of biodegradable polymers unceasingly decreased with increasing the dissolution temperature. Nonetheless, the maximum dissolution temperature for natural biodegradable polymers was 110 °C which can minimize their degradation [14]. Furthermore, the dissolution time also depends on the types of biodegradable polymers and the concentration of biodegradable polymers in ionic liquids.

### 3.2. Ionic Liquids as Additives for Preparing Electrospinning Solutions

Table 6 exhibits ionic liquids, biodegradable polymers, solvents, biodegradable polymer concentrations, dissolving temperatures, and dissolving times that were applied for the preparation of electrospinning solutions. It can be perceived that imidazolium-based ionic liquids with tetrafluoroborate, chloride, and hexafluorophosphate counter anions are typically utilized as additives for the preparation of biodegradable polymer electrospinning solutions. Moreover, natural biodegradable polymers (except for their derivatives) are rarely added with ionic liquids in their electrospun fibers, compared to synthetic biodegradable polymers. Additionally, PLA, PVA, and PHB can be added with ionic liquids after dissolving them in conventional solvents during the preparation of electrospinning solutions [3,34,39]. Moreover, organic solvents are frequently used to dissolve synthetic biodegradable polymers and natural biodegradable polymer derivatives before the addition of ionic liquids. Besides this, the mixture of solvents, for example, acetone/dimethylacetamide and chloroform/methanol have been employed for dissolving a cellulose derivative such as cellulose acetate [24,25,26]. On the other hand, PVA, starch, and gelatin can be dissolved in a polar solvent, such as water [33,34].

In Table 6, it can also be seen that different concentrations of biodegradable polymers have been applied for preparing electrospinning solutions. The highest concentration of cellulose acetate in the mixture of solvents for an electrospinning solution was 17% wt.% [25,26], and the lowest was 8.2 wt.% [24]. In contrast, the highest concentration of PLA in chloroform for an electrospinning solution was 8% wt.% [38], and the lowest was 4.5 wt.% [3]. Instead, the common concentration of PVA in deionized water for an electrospinning solution was 20 wt.% [34,35]. In addition, it can also be noticed in Table 6 that most of the dissolving processes were usually carried out at room temperature. This is presumably caused by the characteristics of most organic solvents, whereby they have a high vapor pressure, which is highly volatile, compared to the ionic liquids that have a negligible vapor pressure, which is non-volatile. Therefore, high temperatures easily evaporate the used solvents into gases. In addition, no specific dissolving times for biodegradable polymers in conventional solvents were stated except for chitosan, PHB, and PLA electrospinning solutions. Furthermore, the addition of ionic liquids as additives can remain steady in biodegradable polymer electrospun fibers for a long period until their complete deterioration.

## 4. Properties of Ionic Liquid-Assisted Electrospun Biodegradable Polymer Fibers

### 4.1. Properties of Electrospun Biodegradable Polymer Fibers Assisted by Ionic Liquids as Solvents

**Figure 5:**
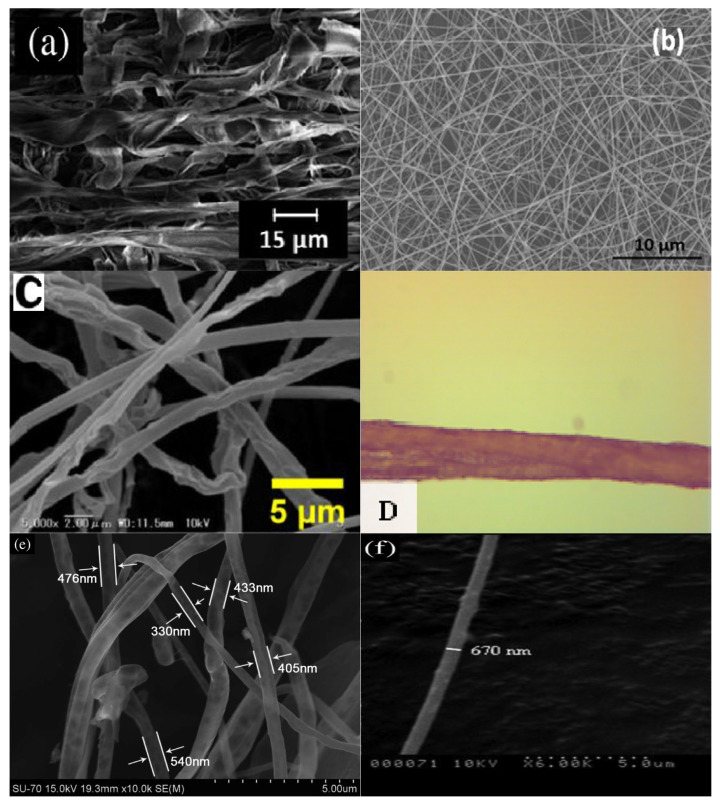
SEM micrographs of (**a**) electrospun cellulose fibers [6]; (**b**) electrospun silk fibers [36]; (**c**) electrospun cellulose fibers [14]; (**d**) electrospun chitin/PLA blend fiber [29]; (**e**) electrospun cellulose fibers [7]; and (**f**) electrospun chitin fiber [1] assisted by different ionic liquids as solvents.

**Figure 6:**
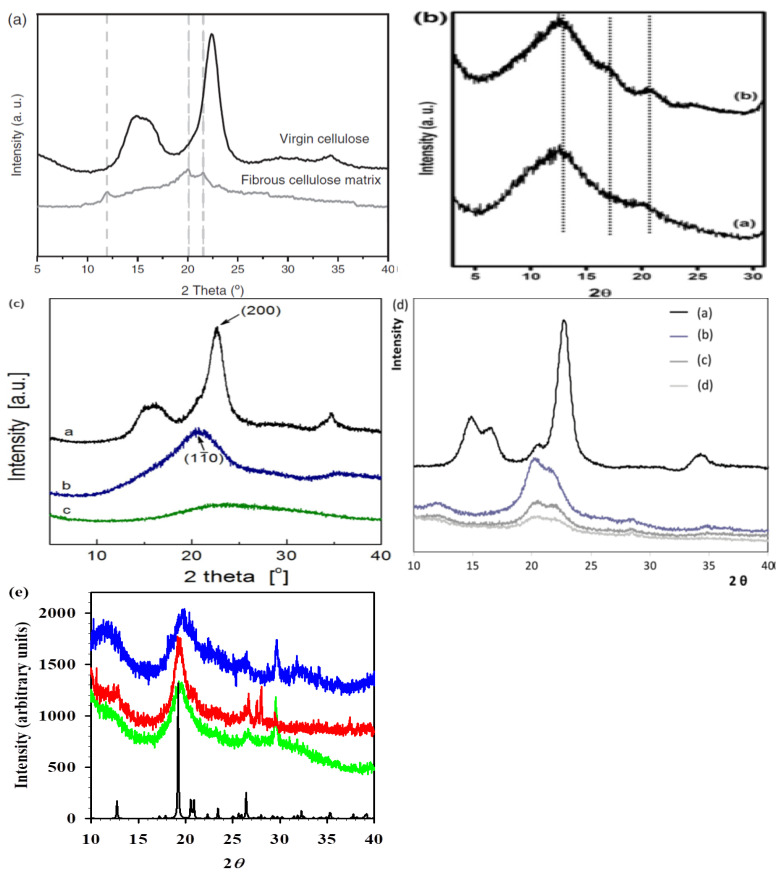
XRD patterns of (**a**) electrospun cellulose fibers (**bottom**) [6]; (**b**) electrospun silk fibers (**top**) [36]; (**c**) electrospun cellulose fibers (**bottom**) [14]; (**d**) electrospun cellulose fibers (middle c) [7]; and (**e**) electrospun chitin fibers (**top**) [1] assisted by different ionic liquids as solvents.

Table 7 shows the properties of electrospun biodegradable polymer fibers were assisted by ionic liquids as solvents. The electrospun cellulose fibers were prepared by Ciuzas et al. from raw cellulose fibers using [C_4_mim][OAc] as a solvent [6]. The morphological, chemical, crystalline, and thermal properties of the prepared electrospun fibers were characterized by a scanning electron microscope (SEM), Fourier transform infrared spectrometer, X-ray diffractometer (XRD), and thermal gravimetric analyzer. The morphological property, such as the average width of the electrospun cellulose fibers is 1.95 ± 0.9 μm with the formation of a continuous ribbon-like structure (Figure 5a). This suggested that the electrospinning process can generate fiber dimensions of almost micrometers. In addition, the chemical property, such as the infrared spectrum of the electrospun cellulose fibers showed almost no difference from the spectrum of the virgin cellulose. This indicated that the cellulose retained its original chemical structure, as well as [C_4_mim][OAc] and the washing solvents which were adequately leached from the electrospun cellulose fibers [6]. Nevertheless, the crystalline property, such as the intensity of the X-ray diffraction peak of the electrospun cellulose fibers is significantly decreased, compared to the virgin cellulose (Figure 6a). This was due to the conversion from cellulose I to cellulose II of the electrospun cellulose fibers. Additionally, the thermal property, such as the decomposition temperature of the electrospun cellulose fibers is lower than that of the virgin cellulose. This implies that the electrospun cellulose fibers have less thermal stability [6]. Therefore, it can be concluded that the use of [C_4_mim][OAc] provides electrospun cellulose fibers with low crystallinity and low thermal stability.

Meanwhile, the electrospun silk fibers were prepared by Srivastava and Purwar from muga silk cocoons using [C_4_mim][OAc] as a solvent [36]. The morphological, chemical, crystalline, and thermal properties of the prepared electrospun fibers were characterized by a scanning electron microscope, Fourier transform infrared spectrometer, X-ray diffractometer, and thermal gravimetric analyzer. The morphological property, such as the average thickness of the electrospun silk fibers is 160 nm with a solid surface and randomly oriented with interconnected pores between the fibers (Figure 5b). This confirmed that the electrospinning process generated silk fibers at the nanoscale level with a porous morphology. In addition, the chemical property, such as the infrared spectrum of the electrospun silk fibers indicated that the [C_4_mim][OAc] vibrational peaks completely disappeared like a silk cast film. This was attributed to the removal of [C_4_mim][OAc] by methanol after the electrospinning process [36]. Moreover, the crystalline property, such as the intensity of the X-ray diffraction pattern of the electrospun silk fibers is almost similar to the diffraction pattern of the silk cast film (Figure 6b). This is because the crystalline structure of electrospun silk fibers is not much different from that of silk cast film. Moreover, the thermal property, such as the degradation temperature of the electrospun silk fibers is nearly unchanged, which is about the same as the silk cast film. This demonstrated that both biodegradable materials have similar thermal stability [36]. Hence, it can be inferred that the utilization of [C_4_mim][OAc] gives electrospun silk fibers a consistent crystallinity and constant thermal stability.

The electrospun cellulose fibers were prepared by Xu et al. from raw cellulose using [C_4_mim][Cl] as a solvent [14]. The morphological, chemical, crystalline, and thermal properties of the prepared electrospun fibers were characterized by a scanning electron microscope, Fourier transform infrared spectrometer, wide angle X-ray diffractometer, and thermal gravimetric analyzer. The morphological property, such as the average thickness of the electrospun cellulose fibers is around 1 μm with a uniform distribution without beads and blocks (Figure 5c). This proved that the thickness of the electrospun fibers is in the microscale dimension. Additionally, the chemical property, such as the infrared spectrum of the electrospun cellulose fibers can be said to be identical to the spectrum of the raw cellulose. This indicated that no obvious chemical reaction occurred during the dissolution of cellulose in [C_4_mim][Cl] [14]. Nonetheless, the crystalline property, such as the intensity of the X-ray diffraction peak of the electrospun cellulose fibers is considerably reduced, compared to the raw cellulose (Figure 6c). This was ascribed to the crystalline form, and the hydrogen bonding networks were destroyed by the ions of [C_4_mim][Cl] during dissolution. In addition, the thermal property, such as the decomposition temperature of the electrospun cellulose fibers decreased in comparison to the raw cellulose. This was caused by a decrease in the crystallinity of the electrospun cellulose fibers [14]. Thus, it can be deduced that the usage of [C_4_mim][Cl] grants electrospun cellulose fibers with low crystallinity and low thermal stability.

The electrospun chitin/PLA blend fibers were prepared by Shamshina et al. from different contents of PLA using [C_2_mim][OAc] as a solvent [29]. The morphological, chemical, and thermal properties of the prepared electrospun blend fibers were characterized by an optical microscope, a Fourier transform infrared spectrometer, and a thermal gravimetric analyzer. The morphological property, such as the average thickness of the electrospun chitin/PLA blend fibers increased with an increasing PLA content (up to 172 ± 33 μm) with non-uniform blend fiber thicknesses (Figure 5d). This suggested that higher overall concentrations of biodegradable polymers often result in non-uniform thicknesses. Furthermore, PLA can also interfere with the hydrogen bonding network of chitin. However, the chemical property, such as the infrared characteristic peaks of the electrospun chitin/PLA blend fibers shifted to lower wavenumbers, compared to the neat PLA. This verified the presence of intermolecular hydrogen bonding between the carbonyl groups of PLA and the amide groups of chitin in the electrospun-blend fibers [29]. Moreover, the thermal property, such as the decomposition temperature of the electrospun chitin/PLA blend fibers significantly improved in comparison to the neat chitin, but it also decreased when compared to the neat PLA. This confirmed that the uniform chitin/PLA blend fibers were obtained. Therefore, it can be concluded that the use of [C_2_mim][OAc] provides electrospun chitin/PLA blend fibers with good intermolecular interaction and high thermal stability.

The electrospun cellulose fibers were prepared by Freire et al. from raw cellulose fibers using [C_2_mim][OAc] as a solvent [7]. The morphological, chemical, crystalline, and thermal properties of the prepared electrospun fibers were characterized by a scanning electron microscope, Fourier transform infrared spectrometer, X-ray diffractometer, and thermal gravimetric analyzer. The morphological property, such as the average thickness of the electrospun cellulose fibers is approximately 470 ± 110 nm with moderate and more homogeneous fibers (Figure 5e). This implied that the thickness of the electrospun fibers could be reached the nano-level. Furthermore, the chemical property, such as the infrared spectrum of the electrospun cellulose fibers revealed the absence of new bands. This validated that no traces of acetate anions from the ionic liquid could be detected [7]. On the other hand, the crystalline property, such as the intensity of the X-ray diffraction peak of the electrospun cellulose fibers substantially decreased, compared to the raw cellulose (Figure 6d). This was due to the conversion of cellulose Type-I into cellulose Type-II which was induced by the interruption of the inter- and intramolecular hydrogen bonding during the dissolution of cellulose in [C_2_mim][OAc]. Nevertheless, the thermal property, such as the degradation temperature of the electrospun cellulose fibers is slightly lower than that of raw cellulose, but it is higher in comparison to the cellulose casting film. This demonstrated that the fibrillar morphology of the electrospun fibers contributed to a higher thermal stability [7]. Hence, it can be inferred that the utilization of [C_2_mim][OAc] provides electrospun cellulose fibers with low crystallinity and high thermal stability.

The electrospun chitin fibers were prepared by Barber et al. from dried shrimp shells using [C_2_mim][OAc] as a solvent [1]. The morphological, chemical, and crystalline properties of the prepared electrospun fibers were characterized by a scanning electron microscope, a Fourier transform infrared spectrometer, and an X-ray diffractometer. The morphological property, such as the thickness of the electrospun chitin fibers, is 670 nm with smooth and continuous fibers (Figure 5f). This indicated that the thickness of the electrospun fibers could be achieved on the scale of a submicron. In addition, the chemical property, such as the infrared spectrum of the electrospun chitin fibers is almost identical to the spectrum of the practical grade chitin. This displayed the similarity of their chemical structures and the absence of any significant changes in the functional groups after the electrospinning process [1]. Nonetheless, the crystalline property, such as the intensity of the X-ray diffraction peak of the electrospun chitin fibers, is moderately reduced, compared to the practical grade chitin (Figure 6e). This may be due to a decrease in the crystal structure of the electrospun fibers. Thus, it can be deduced that the usage of [C_2_mim][OAc] grants electrospun chitin fibers with unchanged chemical properties and low crystallinity.

### 4.2. Properties of Electrospun Biodegradable Polymer Fibers Assisted by Ionic Liquids as Additives

**Figure 7:**
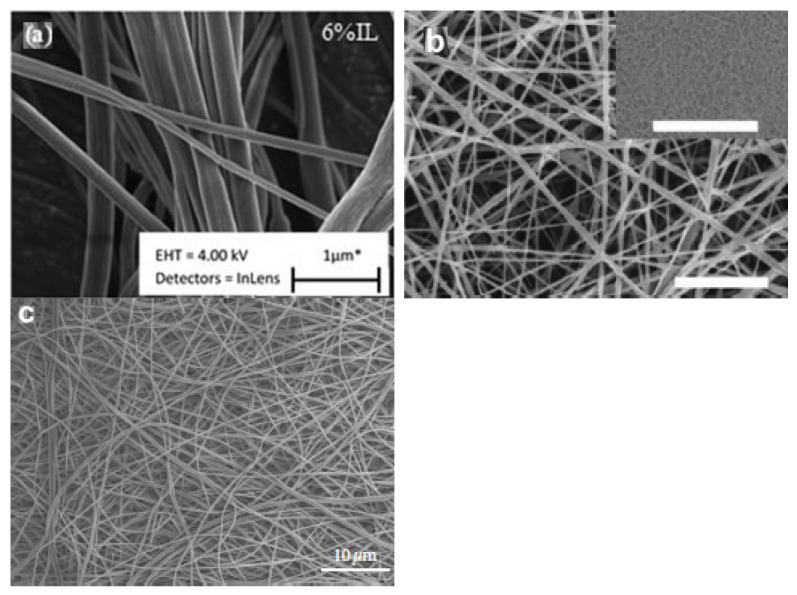
SEM micrographs of (**a**) electrospun cellulose acetate fibers [25]; (**b**) electrospun PLA fibers [39]; and (**c**) electrospun gelatin fibers [33] assisted by different ionic liquids as additives.

**Figure 8:**
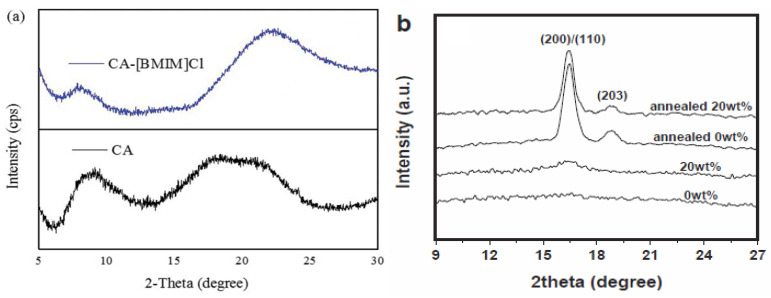
XRD patterns of (**a**) electrospun cellulose acetate fibers (**top**) [25]; and (**b**) electrospun PLA fibers (**top**) [39] assisted by different ionic liquids as additives.

Table 8 illustrates the properties of electrospun biodegradable polymer fibers that are assisted by ionic liquids as additives. The electrospun cellulose acetate fibers were prepared by Javed et al. from cellulose acetate powder using [C_4_mim][Cl] as an additive [25,26]. The morphological, chemical, crystalline, and thermal properties of the prepared electrospun fibers were characterized by a scanning electron microscope, Fourier transform infrared spectrometer, X-ray diffractometer, and thermal gravimetric analyzer. The morphological property, such as the average thickness of the electrospun cellulose acetate-[C_4_mim][Cl] fibers increased by up to 525 nm with an addition of 6% of [C_4_mim][Cl] (Figure 7a), compared to the electrospun cellulose acetate fibers (125 nm). This was attributed to the lower viscoelastic force that was created by reducing the concentration of [C_4_mim][Cl] during the electrospinning process, which reduced the stretching force. Besides this, the chemical property, such as the infrared characteristic bands of the electrospun cellulose acetate-[C_4_mim][Cl] fibers shifted to higher wavenumbers in comparison to the electrospun cellulose acetate fibers. This suggested that the cations and anions of [C_4_mim][Cl] formed hydrogen bonding with cellulose acetate [25]. However, the crystalline property, such as the intensity of the X-ray diffraction peak of the electrospun cellulose acetate-[C_4_mim][Cl] fibers considerably decreased, compared to the electrospun cellulose acetate fibers (Figure 8a). This implied a reduction in crystallinity because of the disruption of the molecular packing of the cellulose acetate by [C_4_mim][Cl]. Moreover, the thermal property, such as the decomposition temperature of the electrospun cellulose acetate-[C_4_mim][Cl] fibers is lower than the electrospun cellulose acetate fibers. This demonstrated that the electrospun cellulose acetate-[C_4_mim][Cl] fibers have less thermal stability [26]. Therefore, it can be concluded that the addition of [C_4_mim][Cl] into cellulose acetate provides electrospun fibers with low crystallinity and low thermal stability.

Meanwhile, the electrospun PLA fibers were prepared by Na et al. from poly (L-lactide) using [C_2_mim][NTf_2_] as an additive [39]. The morphological, chemical, crystalline, and thermal properties of the prepared electrospun fibers were characterized by a scanning electron microscope, Fourier transform infrared spectrometer, X-ray diffractometer, and differential scanning calorimeter. The morphological property, such as the average thickness of the electrospun PLA-[C_2_mim][NTf_2_] fibers decreased to 72.1 ± 34.2 nm with an addition of 20 wt.% of [C_2_mim][NTf_2_] (Figure 7b), compared to the electrospun PLA fibers (394.8 ± 150.9 nm). This displayed that the presence of [C_2_mim][NTf_2_] formed nano-sized electrospun PLA fibers. Moreover, the addition of [C_2_mim][NTf_2_] substantially increased the conductivity and simultaneously decreased the viscosity of the PLA solution, which affected the solution stretching and consequently lessened the thickness of the electrospun fibers. Furthermore, the chemical property, such as the infrared absorption band of the electrospun PLA-[C_2_mim][NTf_2_] fibers (20 wt.%) shifted to a lower wavenumber in comparison to the electrospun PLA fibers. This was ascribed to the PLA mesophase with a certain degree of molecular arrangement caused by an exceptional stretching of the PLA solution during electrospinning [39]. Nonetheless, the crystalline property, such as the intensity of the X-ray diffraction peak of the electrospun PLA-[C_2_mim][NTf_2_] fibers (20 wt.%) marginally reduced compared to the electrospun PLA fibers (Figure 8b). This indicated that the degree of crystallization was slight, owing to the stress crystallization in the PLA fibers. In addition, the thermal property, such as the melting point of the electrospun PLA-[C_2_mim][NTf_2_] fibers is higher than electrospun PLA fibers. This was because the fibrillar crystals were produced through the mesophase transformation with unusual molecular alignment during cold crystallization [39]. Hence, it can be inferred that the addition of [C_2_mim][NTf_2_] into PLA gives electrospun fibers low crystallinity and a high melting point.

The electrospun gelatin fibers were prepared by Kotatha et al. from bovine skin powder using [C_2_mim][BF_4_] as an additive [33]. The morphological, chemical, and thermal properties of the prepared electrospun fibers were characterized by a scanning electron microscope, a Fourier transform infrared spectrometer, and a thermal gravimetric analyzer. The morphological property, such as the structural morphology of the electrospun gelatin-[C_2_mim][BF_4_] fibers is stable and not deformed after immersion in [C_2_mim][BF_4_] at room temperature for 10 days, with an average thickness size of 428.4 nm (Figure 7c). This revealed that the electrospun gelatin fibers could serve as a suitable host for [C_2_mim][BF_4_]. Additionally, the chemical property, such as the infrared spectrum of the electrospun gelatin-[C_2_mim][BF_4_] fibers showed the presence of new characteristic peaks of [C_2_mim][BF_4_]. This confirmed that [C_2_mim][BF_4_] attached to the electrospun gelatin fibers [33]. Moreover, the thermal property, such as the degradation temperature of the electrospun gelatin-[C_2_mim][BF_4_] fibers significantly improved compared to the electrospun gelatin fibers. This demonstrated that the electrospun gelatin-[C_2_mim][BF_4_] fibers have more thermal stability. Thus, it can be deduced that the addition of [C_2_mim][BF_4_] into gelatin grants electrospun fibers with a stable fibrous structure and high thermal stability.

## 5. Conclusions

In this paper, examples of natural and synthetic biodegradable polymers, ionic liquids that are utilized in the electrospinning process, and the preparations for electrospinning solutions were concisely reviewed. Additionally, the essential properties of electrospun biodegradable polymer fibers, such as their morphological, chemical, crystalline, and thermal properties were also viewed in this concise review. From preceding studies, it was found that ionic liquids that are utilized as solvents for the dissolution of biodegradable polymers are usually based on imidazolium cations with acetate and chloride counter anions. On the other hand, ionic liquids that are utilized as additives in electrospinning solutions are typically based on imidazolium cations with tetrafluoroborate, chloride, and hexafluorophosphate counter anions. Further, the utilization of ionic liquids as solvents generates electrospun biodegradable polymer fibers with average thicknesses from micrometers to nanometers. In addition, ionic liquids that are utilized as solvents do not change the chemical structures of biodegradable polymers. However, the crystallinity and thermal stability of electrospun fibers decreased compared to their neat biodegradable polymers. Moreover, the utilization of ionic liquids as additives increased the average thicknesses of electrospun biodegradable polymer fibers, and the chemical properties of electrospun fibers changed when ionic liquids were added to biodegradable polymers. Nevertheless, the crystallinity of electrospun fibers reduced in comparison to the electrospun fibers without the addition of ionic liquids. In contrast, the addition of ionic liquids improved the thermal properties of electrospun fibers. Therefore, this concise review might assist not only in the preparation of electrospun biodegradable polymer fibers but also in comparing the properties of electrospun fibers.

## Figures and Tables

**Table 1:** Examples of natural and synthetic biodegradable polymers employed for the preparation of electrospun fibers.

Natural Polymer	References	Synthetic Polymer	Abbreviation	References
Cellulose	[2,5,6,7,8,9,10,11,12,13,14,15,16,17,18,19,20,21,22,23]	*Microbiologically made*		
Cellulose acetate	[24,25,26]	Polyhydroxybutyrate	PHB	[3]
Chitin	[1,9,19,27,28,29]			
Chitosan	[12,30]	*Petroleum-based*		
Collagen	[19]	Polybutylene succinate	PBS	[31]
Ethyl cellulose	[32]	Polycaprolactone	PCL	[4]
Gelatin	[33]	Polyvinyl alcohol	PVA	[34,35]
Heparin	[13]			
Silk	[19,36,37]	*Natural waste*		
Starch	[34]	Polylactic acid	PLA	[3,29,38,39]

**Table 2:** Examples of ionic liquids utilized in the electrospinning of biodegradable polymers.

Ionic Liquid	Abbreviation	References
1-Allyl-3-methylimidazolium chloride	[C_3_mim][Cl]	[2,11]
1-Butyl-3-methylimidazolium acetate	[C_4_mim][OAc]	[6,16,23,36]
1-Butyl-3-methylimidazolium chloride	[C_4_mim][Cl]	[4,13,14,17,19,25,26]
1-Butyl-3-methylimidazolium hexafluorophosphate	[C_4_mim][PF_6_]	[24,39]
1-Butyl-3-methylimidazolium tetrafluoroborate	[C_4_mim][BF_4_]	[30,34,39]
1-Ethyl-3-methylimidazolium acetate	[C_2_mim][OAc]	[1,5,7,8,9,10,12,15,16,18,20,21,22,27,28,29]
1-Ethyl-3-methylimidazolium benzoate	[C_2_mim][PhCO_2_]	[13]
1-Ethyl-3-methylimidazolium bis(trifluoromethylsulfonyl)imide	[C_2_mim][NTf_2_]	[39]
1-Ethyl-3-methylimidazolium chloride	[C_2_mim][Cl]	[37]
1-Ethyl-3-methylimidazolium tetrafluoroborate	[C_2_mim][BF_4_]	[32,33]
1-Decyl-3-methylimidazolium chloride	[C_10_mim][Cl]	[16]
Didecyldimethylammonium nitrate	[DDA][NO_3_]	[3]
1-Hexyl-3-methylimidazolium chloride	[C_6_mim][Cl]	[35,38]

**Table 3:** Examples of ionic liquids utilized as solvents for electrospinning of biodegradable polymers.

Ionic Liquid	Biodegradable Polymer	References
[C_3_mim][Cl]	Cellulose	[2,11]
[C_4_mim][OAc]	Cellulose	[6,16,23]
[C_4_mim][OAc]	Silk	[36]
[C_4_mim][Cl]	Cellulose	[13,14,17,19]
[C_4_mim][Cl]	Silk	[19]
[C_4_mim][Cl]	PCL	[4]
[C_2_mim][OAc]	Cellulose	[5,7,8,9,10,12,15,16,18,20,21,22]
[C_2_mim][OAc]	Chitin	[1,9,27,28,29]
[C_2_mim][OAc]	Chitosan	[12]
[C_2_mim][OAc]	PLA	[29]
[C_2_mim][PhCO_2_]	Heparin	[13]
[C_2_mim][Cl]	Silk	[37]
[C_10_mim][Cl]	Cellulose	[16]

**Table 4:** Examples of ionic liquids utilized as additives for electrospinning of biodegradable polymers.

Ionic Liquid	Biodegradable Polymer	References
[C_4_mim][Cl]	Cellulose acetate	[25,26]
[C_4_mim][PF_6_]	Cellulose acetate	[24]
[C_4_mim][PF_6_]	PLA	[39]
[C_4_mim][BF_4_]	PLA	[39]
[C_4_mim][BF_4_]	Chitosan	[30]
[C_4_mim][BF_4_]	PVA	[34]
[C_4_mim][BF_4_]	Starch	[34]
[C_2_mim][NTf_2_]	PLA	[39]
[C_2_mim][BF_4_]	Ethyl cellulose	[32]
[C_2_mim][BF_4_]	Gelatin	[33]
[DDA][NO_3_]	PHB	[3]
[DDA][NO_3_]	PLA	[3]
[C_6_mim][Cl]	PLA	[38]
[C_6_mim][Cl]	PVA	[35]

**Table 5:** Ionic liquids, biodegradable polymers, biodegradable polymer concentrations, dissolution temperatures, and dissolution times applied for the preparation of electrospinning solutions.

Ionic Liquid	Biodegradable Polymer	Concentration (wt.%)	Temperature (°C)	Time (Hour)	References
[C_3_mim][Cl]	Cellulose	5	80	2	[2]
[C_4_mim][OAc]	Cellulose	3	90	72	[6,16,23]
[C_4_mim][OAc]	Silk	10	95	2	[36]
[C_4_mim][Cl]	Cellulose	10	70	0.03	[13]
[C_4_mim][Cl]	Cellulose	9.1	110	2	[14]
[C_4_mim][Cl]	Cellulose	5	80	0.5	[17]
[C_4_mim][Cl]	Cellulose	10	100	U	[19]
[C_4_mim][Cl]	Silk	5	100	U	[19]
[C_4_mim][Cl]	PCL	20	U	12	[4]
[C_2_mim][OAc]	Cellulose	1.2	80	8	[5]
[C_2_mim][OAc]	Cellulose	8	25	72	[7]
[C_2_mim][OAc]	Cellulose	1.75	80	U	[8,18]
[C_2_mim][OAc]	Cellulose	1.55	80	8	[15]
[C_2_mim][OAc]	Cellulose	2.5	80	12	[22]
[C_2_mim][OAc]	Chitin	0.4	100	12	[9]
[C_2_mim][OAc]	Chitin	0.5	90	8	[28]
[C_2_mim][OAc]	Chitin	0.45	U	0.03	[1]
[C_2_mim][OAc]	Chitin	1.75	100	15	[29]
[C_2_mim][OAc]	Chitosan	0.5	80	1	[12]
[C_2_mim][OAc]	PLA	0.5	100	15	[29]
[C_2_mim][PhCO_2_]	Heparin	2	70	0.03	[13]
[C_2_mim][Cl]	Silk	10	95	U	[37]
[C_10_mim][Cl]	Cellulose	3	90	72	[16]

U = unstated.

**Table 6:** Ionic liquids, biodegradable polymers, solvents, biodegradable polymer concentrations, dissolving temperatures, and dissolving times applied for the preparation of electrospinning solutions.

Ionic Liquid	Biodegradable Polymer	Solvent	Concentration (wt.%)	Temperature (°C)	Time (Hour)	References
[C_4_mim][Cl]	Cellulose acetate	Ace/DMAc	17	R	U	[25,26]
[C_4_mim][PF_6_]	Cellulose acetate	Chl/MeOH	8.2	R	U	[24]
[C_4_mim][PF_6_]	PLA	DCM	U	R	U	[39]
[C_4_mim][BF_4_]	PLA	DCM	U	R	U	[39]
[C_4_mim][BF_4_]	Chitosan	AcOH	3	R	2	[30]
[C_4_mim][BF_4_]	PVA	DIW	20	45	U	[34]
[C_4_mim][BF_4_]	Starch	DIW	5	45	U	[34]
[C_2_mim][NTf_2_]	PLA	DCM	U	R	U	[39]
[C_2_mim][BF_4_]	Gelatin	DIW	25	50	U	[33]
[DDA][NO_3_]	PHB	Chl	5	U	0.17	[3]
[DDA][NO_3_]	PLA	Chl	4.5	R	24	[3]
[C_6_mim][Cl]	PLA	Chl	8	R	U	[38]
[C_6_mim][Cl]	PVA	DIW	20	R	U	[35]

Ace = acetone; DMAc = dimethylacetamide; Chl = chloroform; MeOH = methanol; DCM = dichloromethane; AcOH = acetic acid; DIW = deionized water; R = room; and U = unstated.

**Table 7:** Properties of electrospun biodegradable polymer fibers assisted by ionic liquids as solvents.

Ionic Liquid	Biodegradable Polymer	Properties				References
		Morp	Chem	Crys	Ther	
[C_4_mim][OAc]	Cellulose	⇓	⇕	⇓	⇓	[6]
[C_4_mim][OAc]	Silk	⇓	⇕	⇕	⇕	[36]
[C_4_mim][Cl]	Cellulose	⇓	⇕	⇓	⇓	[14]
[C_2_mim][OAc]	Chitin/PLA	⇑	⇑	-	⇑	[29]
[C_2_mim][OAc]	Cellulose	⇓	⇕	⇓	⇑	[7]
[C_2_mim][OAc]	Chitin	⇓	⇕	⇓	-	[1]

Morp = morphological; Chem = chemical; Crys = crystalline; and Ther = thermal. The symbol ‘⇓’ corresponds to a decrease in the properties, and ‘⇑’ corresponds to an increase in the properties, while ‘⇕’ and ‘-’ describe unchanged and not available, respectively.

**Table 8:** Properties of electrospun biodegradable polymer fibers assisted by ionic liquids as additives.

Ionic Liquid	Biodegradable Polymer	Properties				References
		Morp	Chem	Crys	Ther	
[C_4_mim][Cl]	Cellulose acetate	⇑	⇑	⇓	⇓	[25,26]
[C_2_mim][NTf_2_]	PLA	⇓	⇑	⇓	⇑	[39]
[C_2_mim][BF_4_]	Gelatin	⇕	⇑	-	⇑	[33]

Morp = morphological; Chem = chemical; Crys = crystalline; and Ther = thermal. The symbol ‘⇑’ corresponds to an increase in the properties, and ‘⇓’ corresponds to a decrease in the properties, while ‘⇕’ and ‘-’ describe unchanged and not available, respectively.

## Data Availability

Not applicable.
